# Risk factors and outcome of COVID-19 in patients with hematological malignancies

**DOI:** 10.1186/s40164-020-00177-z

**Published:** 2020-08-25

**Authors:** José Luis Piñana, Rodrigo Martino, Irene García-García, Rocío Parody, María Dolores Morales, Gonzalo Benzo, Irene Gómez-Catalan, Rosa Coll, Ignacio De La Fuente, Alejandro Luna, Beatriz Merchán, Anabelle Chinea, Dunia de Miguel, Ana Serrano, Carmen Pérez, Carola Diaz, José Luis Lopez, Adolfo Jesús Saez, Rebeca Bailen, Teresa Zudaire, Diana Martínez, Manuel Jurado, María Calbacho, Lourdes Vázquez, Irene Garcia-Cadenas, Laura Fox, Ana I. Pimentel, Guiomar Bautista, Agustin Nieto, Pascual Fernandez, Juan Carlos Vallejo, Carlos Solano, Marta Valero, Ildefonso Espigado, Raquel Saldaña, Luisa Sisinni, Josep Maria Ribera, Maria Jose Jimenez, Maria Trabazo, Marta Gonzalez-Vicent, Noemí Fernández, Carme Talarn, Maria Carmen Montoya, Angel Cedillo, Anna Sureda, Infectious Complications Subcommittee of the Spanish Hematopoietic Stem Cell Transplantation and Cell Therapy Group (GETH)

**Affiliations:** 1 Hematology División, Hospital Universitario y Politécnico La Fe, Instituto de Investigación Sanitaria La Fe, Valencia, Spain; 2 CIBERONC, Instituto Carlos III, Madrid, Spain; 3 Hematology División, Hospital de la Santa Creu i Sant Pau, Barcelona, Spain; 4 Hematology División, Hospital Ramon y Cajal, Madrid, Spain; 5 Hematology División, Institut Català Oncologia-Hospital Duran i Reynals, Barcelona, Spain; 6 Hematology División, Hospital de Guadalajara, Guadalajara, Spain; 7 Hematology División, Hospital La Princesa, Madrid, Spain; 8 Hematology División, Hospital de Albacete, Albacete, Spain; 9 Hematology División, Institut Català Oncologia-Hospital Josep Trueta, Girona, Spain; 10 Hematology División, Hospital Clínico de Valladolid, Valladolid, Spain; 11 Hematology División, Hospital Carlos Haya, Malaga, Spain; 12 Hematology División, Hospital Fundación Jiménez Díaz, Madrid, Spain; 13 Hematology División, Hospital Gregorio Marañon, Madrid, Spain; 14 Hematology División, Hospital de Navarra, Navarra, Spain; 15 Hematology División, Hospital a Coruña, Coruña, Spain; 16 Hematology División, Hospital Virgen de la Nieves, Granada, Spain; 17 Hematology División, Hospital 12 de Octubre, Madrid, Spain; 18 Hematology División, Hospital Universitario de Salamanca, Salamanca, Spain; 19 Hematology División, Hospital Vall d`Hebron, Barcelona, Spain; 20 Hematology División, Hospital Clínico Universitario Lozano Blesa, IIS Aragon, Zaragoza, Spain; 21 Hematology División, Hospital Puerta de Hierro, Madrid, Spain; 22 Hematology División, Hospital de Vigo, Vigo, Spain; 23 Hematology División, Hospital General de Alicante, Alicante, Spain; 24 Hematology División, Hospital de Donostia, Donostia, Spain; 25 Hematology División, Hospital Clínico Universitario de Valencia, Valencia, Spain; 26 Hematology División, Hospital Arnau de Vilanova, Valencia, Spain; 27 Department of Hematology, University Hospital Virgen del Rocío/University of Sevilla, CSIC/Institute of Biomedicine of Sevilla, Sevilla, Spain; 28 Hematology División, Hospital de Jerez, Jerez, Spain; 29 Pediatric Hematology-Oncology División, Hospital la Paz, Madrid, Spain; 30 Hematology División, ICO-Hospital Germans Trias i Pujol, Josep Carreras Research Institute, Badalona, Spain; 31 Pediatric División, Hospital de la Santa Creu i Sant Pau, Barcelona, Spain; 32 Pediatric División, Hospital niño Jesús, Madrid, Spain; 33 Hematology División, Hospital Marqués de Valdecilla, Santander, Spain; 34 Hematology División, Hospital Joan XXIII, Tarragona, Spain; 35 Hematopoietic Stem Cell Transplantation and Cell Therapy Group (GETH), Madrid, Spain; 36 Division of Clinical Hematology, Hospital Universitario la Fe de Valencia, Avda Fernando Abril Martorell, 106 CP 46026 Valencia, Spain

## Abstract

**Background:**

Prognostic factors of poor outcome in patients with hematological malignancies and COVID-19 are poorly defined.

**Patients and methods:**

This was a Spanish transplant group and cell therapy (GETH) multicenter retrospective observational study, which included a large cohort of blood cancer patients with laboratory-confirmed SARS-CoV-2 infection through PCR assays from March 1st 2020 to May 15th 2020.

**Results:**

We included 367 pediatric and adult patients with hematological malignancies, including recipients of autologous (ASCT) (n = 58) or allogeneic stem cell transplantation (allo-SCT) (n = 65) from 41 hospitals in Spain. Median age of patients was 64 years (range 1–93.8). Recipients of ASCT and allo-SCT showed lower mortality rates (17% and 18%, respectively) compared to non-SCT patients (31%) (p = 0.02). Prognostic factors identified for day 45 overall mortality (OM) by logistic regression multivariate analysis included age > 70 years [odds ratio (OR) 2.1, 95% confidence interval (CI) 1.2–3.8, p = 0.011]; uncontrolled hematological malignancy (OR 2.9, 95% CI 1.6–5.2, p < 0.0001); ECOG 3–4 (OR, 2.56, 95% CI 1.4–4.7, p = 0.003); neutropenia (< 0.5 × 10^9^/L) (OR 2.8, 95% CI 1.3–6.1, p = 0.01); and a C-reactive protein (CRP) > 20 mg/dL (OR 3.3, 95% CI 1.7–6.4, p < 0.0001). In multivariate analysis of 216 patients with very severe COVID-19, treatment with azithromycin or low dose corticosteroids was associated with lower OM (OR 0.42, 95% CI 0.2–0.89 and OR 0.31, 95% CI 0.11–0.87, respectively, p = 0.02) whereas the use of hidroxycloroquine did not show significant improvement in OM (OR 0.64, 95% CI 0.37–1.1, P = 0.1).

**Conclusions:**

In most patients with hematological malignancies COVID-19 mortality was directly driven by older age, disease status, performance status, as well as by immune (neutropenia) parameters and level of inflammation (high CRP). Use of azithromycin and low dose corticosteroids may be of value in very severe COVID-19.

## Background

The coronavirus infectious disease 2019 (COVID-19) pandemic caused by the new zoonotic coronavirus (SARS-CoV-2) is causing a massive impact globally. Mortality can be as high as 15% in elderly patients, and/or in patients with comorbidities [1, 2]. Risk factors for COVID-19 severity and death include older age, diabetes, hypertension, or cardiac disease [1–4]. Prior experience with seasonal community-acquired respiratory virus (CARV) infections showed that, in immunocompromised patients, these infections are notable for prolonged viral shedding, higher rates of pneumonia and mortality [5, 6]. Thus, it would be expected that COVID-19 be particularly life threatening in patients with hematological malignancies. In fact, initial reports suggested that patients with cancer had an estimated two-fold increased risk of contracting SARS-CoV-2 than the general population and, if infected, also had a higher risk of severe events [intensive care unit (ICU) admission, invasive ventilation, or death] compared to patients without cancer [7–9]. The outcomes of COVID-19 in patients with hematological disorders such as leukemia, lymphoma, myeloma and recipients of autologous (ASCT) or allogeneic hematopoietic stem cell transplantation (allo-SCT) are of utmost interest due to their high degree of humoral and cellular immunosuppression status. Recent studies have reported an overall COVID-19 related mortality of 32 to 40% in hematological patients [10–14]. Future challenges include the identification of prognostic factors that could help in risk assessment and decision-making for effective supportive care and antiviral therapy or in cases of limited access to ICU.

The current study addresses the COVID-19 clinical course, outcome and risk factors for severe disease and mortality in a large series of patients with hematological disorders, including recipients of ASCT and allo-SCT.

## Patients and methods

### Study population

This is a retrospective multicenter cohort study of the Infectious Complications Subcommittee (GRUCINI) of the Spanish Hematopoietic Stem Cell Transplantation and Cell Therapy Group (GETH).

### Inclusion criteria and data preparation

This series included patients (pediatric and adult) with PCR-documented SARS-CoV-2 infection diagnosed from March 1st 2020 to May 15th 2020 in 41 participating Spanish centers. The status of all patients (and thus the study database) was updated on May 21th 2020. During the study period, hematological patients from participating centers with COVID-19 were prospectively registered through REDcap on-line platform in the GETH database by completing an essential medical data form, and more detailed baseline and outcome data were retrospectively requested afterwards with follow-up forms. The information collected included respiratory symptoms (rhinorrhea, cough, dyspnea, oxygen requirement, sinusitis, otitis, and fever), SARS-COV-2-related hospital admission, oxygen requirement, ICU admission, antiviral COVID-19 therapy given for at least 3 consecutive days, corticosteroid use and anti-cytokine therapy. The dose of corticosteroids was divided in 2 groups, ≤ or > 0.5 mg/kg/day of methylprednisolone (or equivalent doses of prednisone, dexamethasone or hydrocortisone). Details on the underlying disease and its treatment(s) were also captured (details not shown). Baseline laboratory variables, if available [absolute lymphocyte and neutrophil counts, C-reactive protein (CRP), IL6, ferritin and D-dimer levels], were also requested at the time or within 3 days after SARS-CoV-2 detection. Detailed microbiological findings and radiological pulmonary patterns were also required for each episode.

### Definitions

We classified COVID-19 stages according to the recent published staging proposal [15]. Briefly, stage 1 refers to early establishment of disease (symptomatic infection of the upper respiratory tract only, with or without fever and generalized malaise). In stage 1 we included patients reported as being asymptomatic by the registering physician, as well as patients with upper respiratory symptoms (rhinorrhea, sinusitis, otitis, or pharyngitis) and/or systemic symptoms (fever, diarrhea, nausea or vomiting, fatigue and myalgia) in the absence of lower respiratory tract disease (LRTD) symptoms and/or any indication of pulmonary infiltrates by radiology, either chest X-ray or computed tomography (CT) scan. Stage II was divided into IIA and IIB. Stage IIA refers to patients with LRTD with radiological proof of pulmonary involvement but without requirements for oxygen support to maintain an oxygen saturation > 92%. Stage IIB included patients meeting the IIA criteria but who required oxygen support (i.e., patients with acute respiratory failure). Finally, we did not classify cases as stage III since we did not have sufficient inflammatory blood markers in all cases, which are required for upgrading a stage IIB to a stage III. Patients with stage IIB thus comprise all patients with severe COVID-19, while stage IIA can be considered to have moderate COVID-19. We considered very severe COVID-19 those who developed stage IIB and those who required ICU admission due to respiratory failure and/or hemodynamic instability.

Disease status at the time of SARS-CoV-2 detection was defined according to each specific disease’s revised criteria for leukemia, myeloproliferative neoplasm, multiple myeloma and lymphoma [16–18]. Performance status at the time of COVID-19 was graded according to the Eastern Cooperative Oncology Group (ECOG) [19]. Cardiomyopathy was defined by the patient’s medical history or when the left ventricular ejection fraction was < 50%, as were moderate to severe valvular disease, prior or current history of coronary artery disease, or heart failure.

### Technical and diagnostic considerations

Patients with URTD and/or LRTD symptoms underwent nasopharyngeal aspiration, nasopharyngeal swabs, or an induced sputum test, while BAL was performed very rarely and only in patients with negative PCR in the upper airway with radiology-proven LRTD and whenever an alternative diagnosis for the patient was deemed possible by each treating team. Most patients underwent weekly PCR test monitoring until negativity of SARS-CoV-2, especially when the patients improved and discharge was being considered, or for epidemiological reasons. The specific method used for performing the PCR was not captured. During this pandemic, GETH published on-line recommendations on the diagnosis, management, testing and infectious control measures (available in the following website; https://www.geth.es/).

### Endpoints and statistical analysis

The primary objective of the study was to describe clinical characteristics of COVID-19 in onco-hematological patients. We also analyzed potential risk factors (RFs) for the development of severe COVID-19 (stage IIB) and day 45 mortality after SARS-CoV-2 detection. Lastly, we explored the effect of antiviral and anti-cytokine therapy on mortality in patients with very severe COVID-19.

The main characteristics of patients were reported by descriptive statistics on the total of the available information. Median and range were used for continuous variables, while absolute and percentage frequencies were used for categorical variables. Univariate and multivariate analyses of clinical, laboratory and therapeutic variables associated with outcomes were calculated using logistic regression models. For multivariate analysis, only variables with parameter estimates showing a p value ≤ 0.10 in the univariate analysis were finally included. Two-sided exact P values were reported and p values ≤ 0.05 were considered statistically significant. COVID-19 related mortality according to different clinical and biological variables was estimated from time of SARS-CoV-2 detection using Kaplan–Meier curves and univariate comparisons were made with the log-rank test. All the analyses were performed using the statistical software SPSS v. 20.

## Results

### Patient characteristics

Overall, 388 pediatric and adult patients with hematological malignancies were initially registered. However, 21 cases were excluded due to negative results for SARS-CoV-2 PCR testing, irrespective of being classified as “probable COVID-19” by their hospital or regional epidemiologists based on a compatible clinical presentation and the large numbers of infections in the community. Thus, the study includes 367 patients with laboratory-confirmed SARS-CoV-2. Patient and disease characteristics according to whether they received or not a hematopoietic stem cell transplant (ASCT or allo-SCT) are detailed in Table 1. The median age was 64 years (range 1–93.8). Overall, the most common hematological disease was non-Hodgkin’s lymphoma (n = 91, 25%) followed by plasma cell disorders (n = 81, 22%). As expected, patients who did not receive SCT were older, had been diagnosed more recently of their hematological malignancy and/or had received chemotherapy less than 40 days before the SARS-CoV-2 infection, and thus they also had higher rates of uncontrolled hematological disease (not in partial or complete remission). Of note, they had higher comorbidities such as hypertension, cardiomyopathy and dyslipidemia (p < 0.01 for all comparisons). In contrast, recipients of SCT had received more prior lines of therapy, while allo-SCT recipients were commonly receiving immunosuppressive drugs at the time of COVID-19 diagnosis (p < 0.01 for all comparisons).

**Table 1:** Patient’s characteristics

Characteristics	Hematological disease, non-SCT (n = 244)	ASCT (n = 58)	Allo-SCT (n = 65)	p value
Age (years), median (range)	71 (7–93)	61 (34–75)	48 (1–70)	< 0.0001
0–40 years, n (%)	20 (8)	1 (2)	25 (39)	< 0.0001
41–60 years, n (%)	51 (22)	22 (38)	21 (32)	
61–70 years, n (%)	45 (18)	30 (52)	(17) (26)	
>71 years, n (%)	128 (52)	5 (8)	2 (3)	
Male, n (%)	132 (54)	34 (59)	40 (61)	0.5
Baseline disease, n (%)				< 0.0001
AML	44 (19)	0	23 (35)	
ALL	12 (5)	1 (2)	12 (18)	
MDS	12 (5)	0	10 (15)	
CMPD	27 (11)	0	2 (3)	
NHL	68 (28)	17 (30)	6 (9)	
CLL	2 (10)	0	2 (3)	
Plasmatic cell disorder	40 (16)	38 (66)	3 (5)	
AA or auto-immune disorders	13 (6)	1 (2)	5 (8)	
Disease status, n (%)				< 0.0001
CR/PR	81 (33)/45 (18)	28 (48)/16 (28)	56 (86)/0	
Not in remission (Rel/Ref/Prog)	10 (4)/8 (3)/23 (9)	5 (8)/1 (2)/6 (10)	6 (9)/0/2 (3)	
Active disease not requiring therapy	22 (9)	0	0	
Prior lines of therapy				
0–1	189 (77)	21 (36)	26 (40)	< 0.001
> 1	55 (23)	37 (64)	39 (60)	
Allo-SCT, n (%)				
HLA identical sibling			29 (45)	
Unrelated Donor			22 (34)	
Haplo-identical family donor			14 (21)	
Time from transplant to COVID-19, days (range)		790 (10–10661)	441 (6–7597)	0.1
Prior therapy/conditioning 40 days before COVID-19, n (%)	102 (42)	24 (41)	10 (15)	< 0.0001
Disease diagnosed within 40 days of COVID-19, n (%)	60 (25)	0	0	< 0.0001
Under immunosuppressive drugs before COVID-19				
CNI or sirolimus or MMF	0	0	28 (43)	< 0.0001
Performance status, n (%)				0.2
ECOG 0–1	166 (68)	48 (82)	50 (77)	
ECOG 2/ECOG 3–4	45 (18)/28 (11)	7 (12)/2 (3)	7 (11)/5 (8)	
Pulmonary/cardiovascular risk factors, n (%)				
Active smoking	27 (11)	4 (7)	2 (3)	0.12
Arterial hypertension	118 (48)	12 (21)	12 (18)	< 0.0001
Cardiomyopathy	51 (21)	10 (17)	4 (6)	0.021
Dyslipidemia	78 (32)	11 (19)	5 (8)	< 0.0001
Diabetes	9 (4)	3 (5)	3 (5)	
Median F/U after COVID-19, days (range)	21 (0–74)	30 (0–72)	35 (0–72)	0.2
Median F/U in survivors, days (range)	33 (10–74)	34 (11–72)	40 (12–72)	0.2

SCT: stem cell transplantation; ASCT: autologous stem cell transplantation; allo-SCT: allogeneic hematopoietic stem cell transplantation; AML: acute myeloid leukemia; ALL: acute lymphoblastic leukemia; MDS: myelodysplastic syndrome; cMPD: chronic myeloproliferative disease; NHL: non-Hodgkin lymphoma; CLL: chronic lymphocytic leukemia; AA: aplastic anemia; CR: complete remission; PR: partial remission; Rel: relapse; Ref: refractory; Prog: progression; CNI: calcineurin inhibitors; MMF: mycophenolate mophetil acid; F/U: follow-up

### Clinical characteristics of COVID-19 in non-transplant and transplant patients

**Fig. 1:**
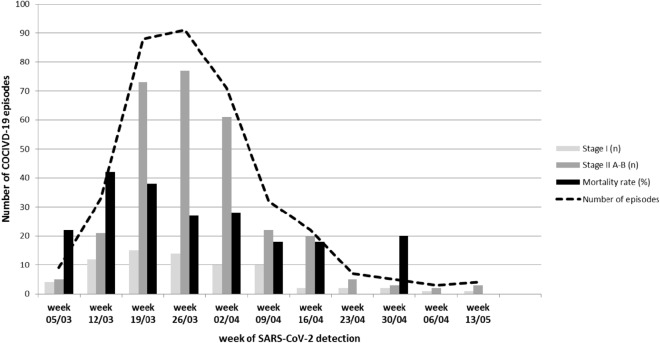
Number of cases and mortality rate according to the severity of COVID-19 and the date of diagnostic

Detailed clinical and laboratory characteristics of COVID-19 by patient category (non-SCT, ASCT and allo-SCT recipients) are shown in Table 2. Most patients (n = 250, 68%) were diagnosed from March 19th to April 8th (see Fig. 1). Out of the 367 cases, 285 (78%) were diagnosed in the outpatient or emergency units, and non-SCT patients were more commonly diagnosed during a hospital admission for treatment of their hematological disease or its complications (23% vs. 12% in SCT recipients).

**Table 2:** Clinical and laboratory characteristics

Characteristics	Hematological disease, non-SCT (n = 244)	ASCT (n = 58)	Allo-HCT (n = 65)	p value
Place of SARS-CoV-2 infection, n (%)				0.1
Outpatient	180 (74)	50 (86)	55 (85)	
Inpatient in specialized hospital	55 (23)	7 (12)	8 (12)	
Hospice institution	9 (4)	1 (2)	2 (3)	
COVID-related hospital admission, n (%)	163 (67)	42 (72)	44 (68)	0.079
Symptoms, n (%)				
Asymptomatic	19 (8)	6 (10)	5 (8)	0.9
Fever	178 (73)	40 (69)	41 (63)	0.6
Rhinorrhea	29 (12)	11 (19)	14 (22)	0.3
Pharyngitis	15 (6)	3 (5)	9 (14)	0.09
Fatigue	140 (57)	24 (41)	32 (49)	0.25
Myalgia	51 (21)	9 (16)	13 (20)	0.38
Cough	175 (72)	30 (52)	39 (60)	0.069
Diarrhea	54 (22)	15 (26)	12 (18)	0.5
Emesis	23 (9)	8 (14)	6 (9)	0.46
COVID-19 stage^a^, n (%)				
Stage 1	41 (17)	15 (26)	18 (28)	0.075
Stage 2A	70 (29)	22 (38)	19 (29)	0.38
Stage 2B	133 (55)	21 (36)	28 (43)	0.027
Oxygen support, n (%)	136 (56)	24 (41)	32 (49)	0.021
Abnormal radiological pulmonary finding, n (%)	200 (82)	44 (76)	46 (71)	0.35
Antiviral COVID-19 therapy, n (%)				0.05
None	49 (20)	16 (28)	13 (20)	
HCQ	15 (6)	0	11 (17)	
HCQ + AZT	36 (15)	5 (9)	8 (12)	
HCQ + AZT + lop/rit	14 (5)	3 (5)	1 (1)	
lop/rit	29 (12)	4 (9)	5 (8)	
HCQ + lop/rit	32 (13)	14 (24)	8 (12)	
AZT + lop/rit	39 (16)	6 (12)	8 (12)	
AZT	24 (10)	5 (7)	7 (11)	
Remdesivir	3 (1)	3 (5)	2 (3)	
Other	4 (2)	3 (5)	3 (5)	
Corticosteroid therapy				0.029
No	132 (54)	40 (69)	45 (69)	
≤0.5 mg/kg/day^b^	29 (12)	7 (12)	9 (14)	
>0.5 mg/kg/day^b^	83 (34)	11 (19)	11 (17)	
Anti-cytokine supportive therapy, n (%)				
Tocilizumab	26 (11)	10 (17)	14 (21)	0.05
Anakinra	10 (4)	3 (5)	5 (8)	
Baricitinib	6 (2)	1 (2)	0	
Laboratory characteristics at the time of SARS-CoV-2 detection				
ANC < 0.5 × 10^9^/L, n (%)	36 (15)	4 (7)	4 (6)	0.089
ALC < 0.5 × 10^9^/L, n (%)	99 (41)	22 (38)	19 (29)	0.27
Platelet count (× 10^9^/L), median (range)	109 (1–1075)	127 (5–410)	115 (10–548)	0.4
< 20 × 10^9^/L	28 (13)	2 (4)	4 (7)	
21–50 × 10^9^/L	27 (12)	7 (14)	9 (15)	
> 50 × 10^9^/L	166 (75)	42 (82)	46 (78)	
CRP > 20 mg/dL (n/evaluable, %)	152/217 (70)	23/48 (48)	25/54 (46)	0.001
IL-6 > 50 pg/mL (n/evaluable, %)	28/70 (40)	6/15 (40)	11/25 (44)	0.9
Ferritin > 500 µg/mL, (n/evaluable, %)	80/104 (77)	14/21 (67)	25/29 (86)	0.4
D dimer > 500 ng/mL (n/evaluable, %)	127/197 (64)	20/42 (48)	25/46 (54)	0.085
Recovery from COVID-19 (n/evaluable, %)	107/218 (49)	42/57 (74)	33/59 (56)	0.001
PCR negativity documented (n/evaluable, %)	49/131 (37)	15/30 (50)	13/27 (48)	0.01
Median time from diagnosis to negativity, days (range)	20 (4–48)	26 (7–53)	26 (7–43)	0.5
Overall mortality, n (%)	80 (33)	12 (21)	13 (20)	0.1
COVID-19 related mortality, n (%)	76 (31)	10 (17)	12 (18)	0.02
Median time from diagnosis to death, days (range)	7.5 (0–38)	13 (0–51)	8 (0–42)	0.18
Admission to the ICU, n (%)	28 (11)	8 (14)	7 (11)	0.8
ICU mortality rate, n (%)	16 (57)	2 (25)	3 (43)	0.2

SCT: stem cell transplantation; ASCT: autologous stem cell transplantation; allo-SCT: allogeneic hematopoietic stem cell transplantation; HCQ: hydroxi-cloroquine; AZT: azithromycin; lop/rit, lopinavir/ritonavir; ANC: absolute neutrophil count; ALC: absolute lymphocyte count; CRP: C-reactive protein; IL: interleukin; PCR: polymerase chain reaction; ICU: intensive care unit

^a^As suggested in Siddiqi et al. [15]

^b^ Refers to dose of IV methylprednisolone or an equivalent dose of another corticosteroid

During follow-up, 30 patients (8%) who were asymptomatic at the time SARS-CoV-2 infection was made did not develop symptoms during follow-up (no differences by patient type). Otherwise, the most common clinical features were fever (n = 259, 71%) cough (n = 244, 66%), fatigue (n = 192, 53%) and diarrhea (n = 81, 22%). COVID-19 was in stage I in 74 cases (20%), stage IIA in 111 patients (30%) and stage IIB in 182 cases (50%). We did not observe significant variations in clinical symptoms among groups. Although pulmonary involvement was not significantly different amongst groups (83%, 74% and 72% of non-SCT, ASCT and allo-SCT, respectively, p = 0.2), non-SCT and allo-SCT patients had higher rates of acute respiratory failure (stage IIB) than ASCT (55% vs. 48% vs. 36%, respectively, p = 0.027).

Regarding the available laboratory data, non-SCT patients had higher rates of severe neutropenia (< 0.5 × 10^9^/mL) and high CRP (> 20 mg/dL) as compared to ASCT and allo-SCT (p < 0.0001), whereas high IL-6, ferritin and D-dimer levels were similar among groups, although these latter laboratory values were available in less than half of the patients (see Table 2).

### Outcome and mortality

The overall median follow-up after SARS-CoV-2 infection was 25 days (range 0-74) for the entire cohort (see Table 1). At the time of the last follow-up, centers reported a higher proportion of full clinical recovery from COVID-19 in ASCT recipients (74%) as compared to allo-SCT (56%) and non-SCT patients (48%) (p < 0.0001). The proportion of documented SARS-CoV-2 PCR negativity during follow-up screening was also higher in SCT patients (ASCT 50% and allo-SCT 48%) as compared to non-SCT (37%) (p = 0.01) without any significant differences in time to negativity (see Table 2).

**Fig. 2:**
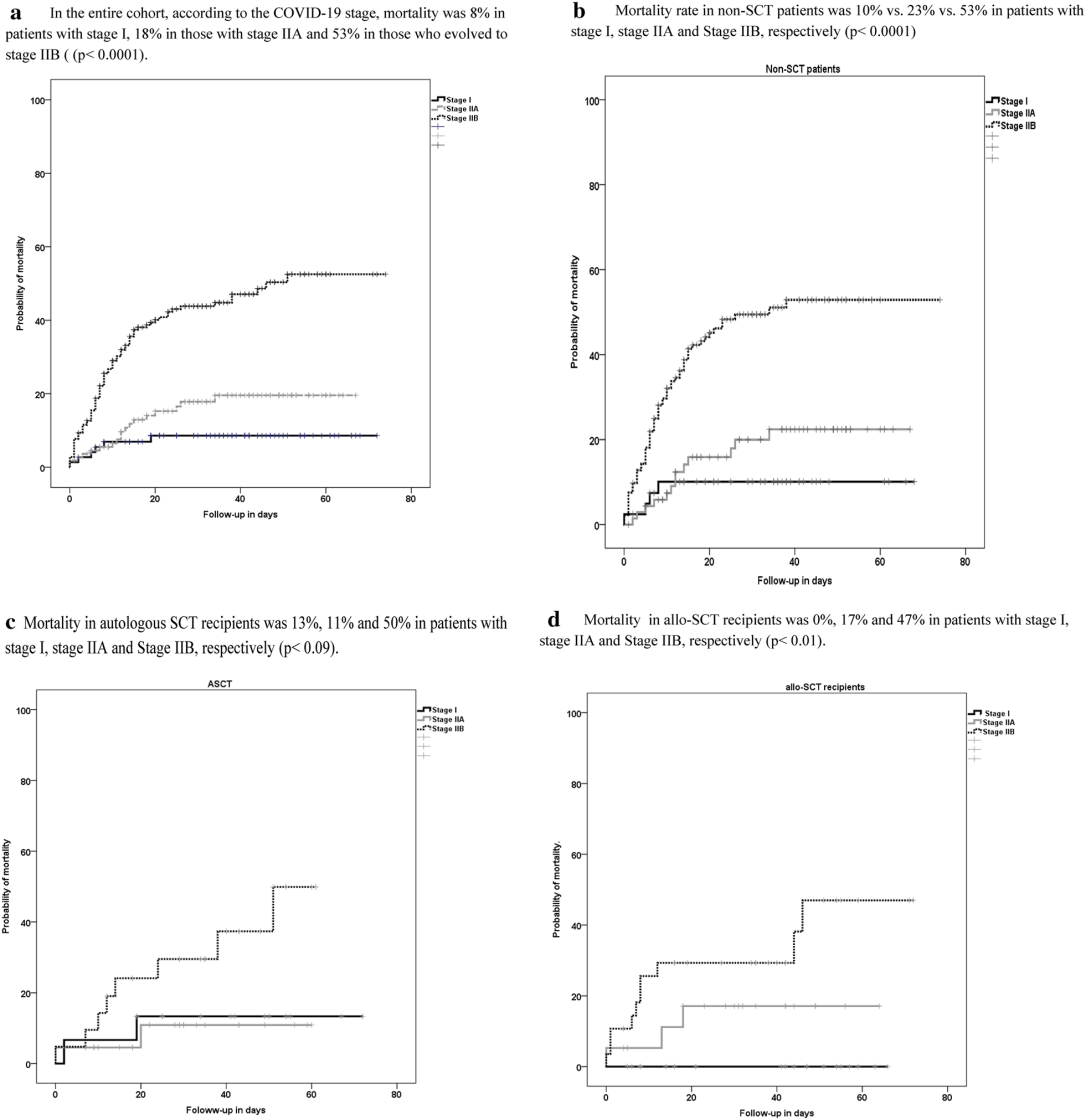
Day 45 overall mortality. **a** In the entire cohort, according to the COVID-19 stage, mortality was 8% in patients with stage I, 18% in those with stage IIA and 53% in those who evolved to stage IIB ((p < 0.0001). **b** Mortality rate in non-SCT patients was 10% vs. 23% vs. 53% in patients with stage I, stage IIA and Stage IIB, respectively (p < 0.0001). **c** Mortality in autologous SCT recipients was 13%, 11% and 50% in patients with stage I, stage IIA and Stage IIB, respectively (p < 0.09). **d** Mortality in allo-SCT recipients was 0%, 17% and 47% in patients with stage I, stage IIA and Stage IIB, respectively (p < 0.01)

Overall mortality at day 45 after SARS-CoV-2 detection was 29% (n = 105), whereas day 45 COVID-19-related mortality was 27% (n = 98). The COVID-19-related mortality was higher in non-SCT (31%) as compared to ASCT (17%) and allo-SCT (18%) (p = 0.02). Median time to death was similar among groups (see Table 2). Overall mortality in patients who were considered suitable candidates for the ICU (n = 43) was high (n = 21, 49%) without differences between groups. Overall mortality rate according to the date of COVID-19 diagnosis is shown in Fig. 1. Overall mortality according to the COVID-19 stage in the entire cohort and by patient groups is shown in Fig. 2a–d.

### Risk factors for severe COVID-19 (stage IIB) and mortality

Logistic regression univariate and multivariate analyses of conditions associated with COVID-19 stage IIB and related mortality in all 367 patients are shown in Table 3.

**Table 3:** Univariate and multivariate analysis of risk factors for stage IIB COVID-19 and COVID-19-related mortality

Variables	Log. Regr. COVID-19 Stage II B^a^ (n = 367)				Log. Regr. COVID-19 Mortality (n = 367)			
	Univariate analysis		Multivariate analysis		Univariate analysis		Multivariate analysis	
	OR (95% CI)	p	OR (95% CI)	p	OR (95% CI)	p	R (95% CI)	p value
Patient age > 70 years old	2.25 (1.45–3.5)	< 0.0001	ns		3.1 (1.9–5)	< 0.0001	2.1 (1.2–3.8)	0.011
Patient age			ns				ns	
0–20	1				1			
21–70	5.6 (1.2–25)	0.02			0.7 (0.22–2.3)	0.59		
>70	11.5 (2.5–52)	0.002			2.3 (0.7–7.5)	0.16		
Sex male	1.41 (0.93–2.1)	0.09	ns		0.95 (0.6–1.5)	0.8		
Baseline disease			ns				ns	
AML	1				1			
ALL	0.8 (0.3–2)	0.68			0.6 (0.22–1.67)	0.3		
MDS	1.3 (0.5–3.4)	0.5			0.69 (0.25–1.9)	0.47		
CMPD	2.7 (1.1–6.8)	0.032			0.6 (0.23–1.5)	0.3		
NHL	1.37 (0.7–2.6)	0.3			0.63 (0.3–1.2)	0.3		
CLL	2.9 (1.12–7.6)	0.03			0.7 (0.35–1.06)	0.06		
Plasmatic cell disorder	0.98 (0.5–1.8)	0.9			0.7 (0.35–1.4)	0.3		
AA or auto-immune disorders	0.3 (0.09–1.1)	0.07			0.08 (0.01–0.69)	0.02		
Disease status			ns					
CR/PR/not requiring therapy	1				1			
Rel/Ref/Prog	1.49 (0.96–2.3)	0.076			3.78 (2.3–6)	< 0.0001	2.9 (1.6–5.2)	< 0.0001
Procedure			ns				ns	
Allo-SCT	1				1			
ASCT	0.75 (0.37–1.5)	0.4			1.04 (0.43–2.5)	0.9		
Non-SCT	1.57 (0.92–2.6)	0.09			1.95 (1.03–3.7)	0.048	ns	
Chemotherapy 40 days before COVID-19	1.18 (0.7–1.8)	0.4	ns		1.76 (1.1–2.79)	0.016	ns	
Disease Dx within 40 days of COVID-19	1.1 (0.6–1.9)	0.7	ns		2.2 (1.24–3.9)	0.007	ns	
ECOG 3–4	1.8 (1.14–2.98)	0.012	ns		3.7 (2.2–6.1)	< 0.0001	2.56 (1.4–4.7)	0.003
Active smoking	1.88 (0.89–3.9)	0.094	ns		1.3 (0.6–2.79)	0.4		
Arterial hypertension	2.26 (1.4–3.49)	< 0.0001	2 (1.3–3.2)	0.002	2.6 (1.6–4.16)	< 0.0001	ns	
Cardiomyopathy	1.98 (1.1–3.4)	0.015	ns		1.6 (0.9–2.88)	0.089	ns	
Dyslipidemia	1.6 (1–2.59)	0.049	ns		1.86 (1.1–3)	0.015	ns	
Place of SARS-CoV-2 infection			ns					
Outpatient	1				1			
Inpatient in specialized hospital	1.1 (0.6–1.8)	0.69			1.05 (0.3–3.6)	0.9		
ALC < 0.5 × 10^9^/L	1.7 (1.1–2.68)	0.014	1.7 (1.1–2.7)	0.015	2.25 (1.6–3.6)	0.001	ns	
ANC < 0.5 × 10^9^/L	1.4 (0.7–2.7)	0.27	ns		3.4 (1.8–6.67)	< 0.0001	2.8 (1.3–6.1)	0.01
Platelet count (× 10^9^/L)							ns	
< 20 × 10^9^/L	1.7 (0.8–3.66)	0.16			4.44 (2.1–9.4)	< 0.0001		
21–50 × 10^9^/L	1.18 (0.6–2.26)	0.6			3.6 (1.8–7.05)	< 0.0001		
> 50 × 10^9^/L	1				1			
CRP > 20 mg/dL	3.1 (1.9–4.9)	< 0.0001	2.67 (1.6–4.3)	< 0.0001	4 (2.2–7.18)	< 0.0001	3.3 (1.7–6.4)	< 0.0001
IL-6 > 50 pg/mL^b^	3.2 (1.38–7.35)	0.007	NT		2.7 (1.1–6.59)	0.028	NT	
Ferritin levels^b^			NT				NT	
<500 µg/mL	1				1			
501–1000 µg/mL	1.18 (0.4–3.2)				1 (0.24–4.1)	0.99		
>1001 µg/mL	2.4 (1.1–5.35)	0.03			2.8 (1–7.9)	0.05		
D dimer > 500 ng/mL^b^	1.6 (1.02–2.6)	0.04	NT		1.34 (0.8–2.3)	0.2	NT	

SCT: stem cell transplantation; ASCT: autologous stem cell transplantation; allo-SCT: allogeneic hematopoietic stem cell transplantation; AML: acute myeloid leukemia; ALL: acute lymphoblastic leukemia; MDS: myelodysplastic syndrome; cMPD: chronic myeloproliferative disease; NHL: non-Hodgkin lymphoma; CLL: chronic lymphocytic leukemia; AA: aplastic anemia; CR: complete remission; PR: partial remission; Rel: relapse; Ref: refractory; Prog: progression; Dx: diagnostic; ANC: absolute neutrophil count; ALC: absolute lymphocyte count; CRP: C-reactive protein; IL: interleukin; ns: not significant; NT: not tested

^a^Stage IIB refers to severe disease, with pulmonary involvement and acute respiratory failure, as suggested in Siddiqi et al. [15]

^b^These variables were not included in the multivariate analyses due to the low number of patients with complete data

By multivariate analysis we identified 3 conditions associated with stage IIB or severe COVID-19; history of hypertension [Odds ratio (OR) 2, 95% confidence interval (CI) 1.3–3.2, p = 0.02], baseline lymphopenia (< 0.5 × 10^9^/mL) (OR 1.7, 95% CI 1.1–2.7, p = 0.015) and baseline CRP > 20 mg/dL (OR 2.67, 95% CI 1.6–4.3, p < 0.0001).

**Fig. 3:**
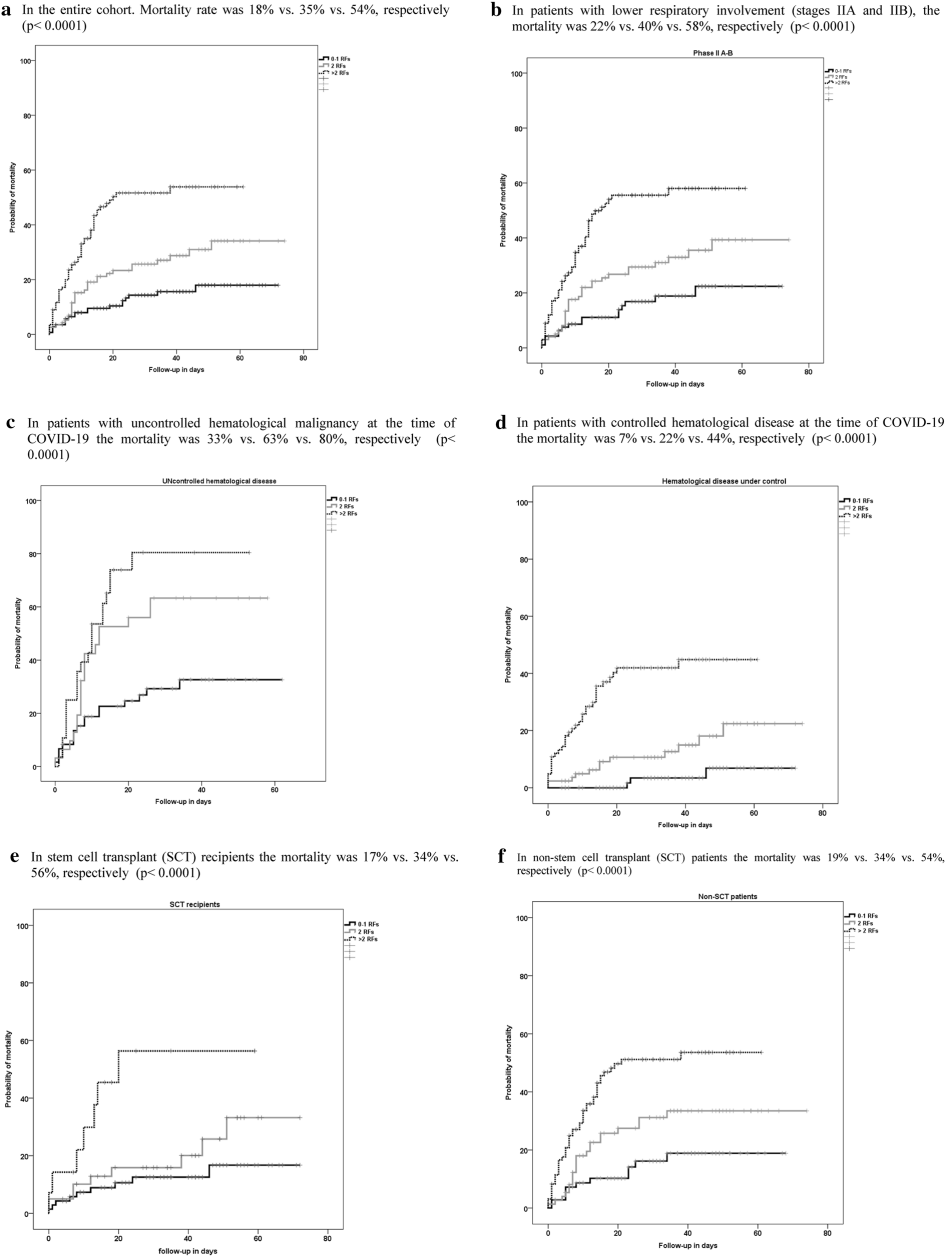
COVID-19-related mortality according to the presence of 0-1, 2 and > 2 risk factors. **a** In the entire cohort. Mortality rate was 18% vs. 35% vs. 54%, respectively (p < 0.0001). **b** In patients with lower respiratory involvement (stages IIA and IIB), the mortality was 22% vs. 40% vs. 58%, respectively (p < 0.0001). **c** In patients with uncontrolled hematological malignancy at the time of COVID-19 the mortality was 33% vs. 63% vs. 80%, respectively (p < 0.0001). **d** In patients with controlled hematological disease at the time of COVID-19 the mortality was 7% vs. 22% vs. 44%, respectively (p < 0.0001). **e** In stem cell transplant (SCT) recipients the mortality was 17% vs. 34% vs. 56%, respectively (p < 0.0001). **f** In non-stem cell transplant (SCT) patients the mortality was 19% vs. 34% vs. 54%, respectively (p < 0.0001)

Finally, 5 conditions were associated with increased COVID-19-related mortality: (i) age > 70 years (OR 2.1, 95% CI 1.2–3.8, p = 0.011); (ii) uncontrolled hematological disease (OR 2.9, 95% CI 1.6–5.2, p < 0.0001); (iii) ECOG 3–4 (OR, 2.56, 95% CI 1.4–4.7, p = 0.003); (iv) neutropenia (< 0.5 × 10^9^/L) (OR 2.8, 95% CI 1.3–6.1, p = 0.01); and (v) CRP > 20 mg/dL (OR 3.3, 95% CI 1.7–6.4, p < 0.0001). Mortality rates progressively increased according to the presence of 0–1, 2 or > 2 of these 5 RFs, as shown in Fig. 3a. Rising mortality by number of RFs was also observed in patients with stages IIA-IIB only, in patients with or without uncontrolled malignancy, and in non-SCT patients or SCT recipients (shown in Fig. 3b–f).

### Effect of anti-viral and anti-cytokine supportive therapy on day 45 COVID-19-related mortality

Most patients (n = 289, 79%) received therapy against SARS-CoV-2, with azithromycin (n = 156, 42%) and hidroxycloroquine (n = 147, 40%) being the most common agents prescribed (details are shown in Table 2). However, each center established its own treatment algorithms, which also probably varied on a patient by patient basis according to the stage and progression of COVID-19. Unfortunately, we did not capture the exact date of start and end of these therapeutic interventions. Thus, in order to evaluate the potential effect of therapy, we performed a second multivariate analysis of COVID-19-related mortality including only the 216 patients (59%) with very severe disease. Results of these univariate and multivariate analyses are shown in Table 4.

**Table 4:** Univariate and multivariate analysis of risk factors for COVID-19-related mortality in patients with very severe COVID-19

Variables	Log. Regr. COVID-19 Mortality in patients with COVID-19 > stage IIA (n = 216)			
	Univariate analysis		Multivariate analysis	
	OR (95% CI) % (95%CI)	p	OR (95% CI)	p
Patient age > 70 years old	2.5 (1.4–4.3)	0.001	2.54 (1.2–5.2)	0.011
Patient age			ns	
0–20	1			
21–70	0.15 (0.017–1.4)	0.11		
> 70	0.45 (0.04–3.9)	0.4		
Sex male	1.2 (0.43–1.3)	0.3		
Baseline disease			ns	
AML	1			
ALL	0.5 (0.13–1.9)	0.31		
MDS	0.48 (0.11–1.4)	0.15		
CMPD	0.25 (0.08–0.76)	0.014		
NHL	0.3 (0.12–0.73)	0.008		
CLL	0.11 (0.03–0.41)	0.001		
Plasmatic cell disorder	0.5 (0.2–1.3)	0.18		
AA or auto-immune disorders	0.09 (0.09–0.89)	0.04		
Disease status				
CR/PR/not requiring therapy	1			
Rel/Ref/Prog	4.3 (2.388–7.8)	< 0.0001	3.5 (1.6–7.5)	0.001
Procedure				
Allo-SCT	1			
ASCT	1.08 (0.38–3)	0.8		
Non-SCT	1.58 (0.74–3.3)	0.23		
Chemotherapy 40 days before COVID-19	1.56 (0.9–2.7)	0.11		
Disease Dx within 40 days of COVID-19	3.5 (1.56–7.8)	0.002	ns	
ECOG 3–4	3.1 (1.7–5.76)	< 0.0001	ns	
Active smoking	0.88 (0.37–2.1)	0.8		
Arterial hypertension	1.97 (1.14–.4)	0.014	2 (1.3–3.2)	0.002
Cardiomyopathy	1.21 (0.6–2.36)	0.56		
Dyslipidemia	1.4 (0.8.–2.6)	0.2		
ALC < 0.5 × 10^9^/L	1.7 (1–3)	0.05	ns	
ANC < 0.5 × 10^9^/L	3.7 (1.58–8.7)	0.002	ns	
Specific therapy				
HCQ	0.64 (0.37–1.1)	0.1	ns	
AZT	0.49 (0.28–0.84)	0.01	0.42 (0.2–0.89)	0.02
HCQ +AZT	0.6 (0.3–1.2)	0.14		
lop/rit	0.7 (0.4–1.2)	0.2		
Remdesivir	0.18 (0.02–1.6)	0.12		
Corticosteroid therapy	0.54 (0.31–0.93)	0.028	ns	
Corticosteroid doses				
No	1		1	
≤0.5 mg/kg/day^b^	0.44 (0.23–0.83)	0.014	0.31 (0.11–0.87)	0.02
>0.5 mg/kg/day^b^	0.54 (0.27–1.06)	0.075	0.75 (0.34–1.6)	0.4
Cytokine inhibitors	0.6 (0.34–1.1)	0.16		
Tocilizumab	0.54 (0.28–1.05)	0.073	ns	
Platelet count (× 10^9^/L)				
≤ 20 × 10^9^/L	6.4 (2–19)	0.001	5.66 (1.44–22)	0.013
21–50 × 10^9^/L	4.1 (1.7–9.7)	0.001	3.3 (1.2–9.2)	0.021
> 50 × 10^9^/L	1		1	
CRP > 20 mg/dL	2.1 (1.08–4.1)	0.027	2.7 (1.1–6.5)	0.029
IL-6 > 50 pg/mL^a^	1.8 (0.69–4.7)	0.2	NT	
Ferritin levels^a^			NT	
<500 µg/mL	1			
501–1000 µg/mL	0.9 (0.2–4.3)	0.9		
>1001 µg/mL	2.2 (0.72–7)	0.15		
D dimer > 500 ng/mL^a^	1.39 (0.76–2.6)	0.2	NT	

SCT: stem cell transplantation; ASCT: autologous stem cell transplantation; allo-SCT: allogeneic hematopoietic stem cell transplantation; AML: acute myeloid leukemia; ALL: acute lymphoblastic leukemia; MDS; myelodysplastic syndrome; cMPD: chronic myeloproliferative disease; NHL: non-Hodgkin lymphoma; CLL: chronic lymphocytic leukemia; AA: aplastic anemia; CR: complete remission; PR: partial remission; Rel: relapse; Ref: refractory; Prog: progression; Dx: diagnostic; ANC: absolute neutrophil count; ALC: absolute lymphocyte count; HCQ: hydroxi-cloroquine; AZT: azithromycin; lop/rit: lopinavir/ritonavir; CRP: C-reactive protein; IL: interleukin; ns: not significant; NT: not tested

^a^These variables were not included in the multivariate analyses due to the low number of patients with complete data

^b^Refers to dose of IV methylprednisolone or an equivalent dose of another corticosteroid

Treatments which led to lower mortality in multivariate analysis were (i) the use of azithromycin (OR 0.42, 95% CI 0.2–0.89, p = 0.02), and (ii) the use of corticosteroids at doses ≤ 0.5 mg/kg/day (OR 0.31, 95% CI 0.11–0.87, p = 0.02); higher doses of corticosteroids (> 0.5 mg/kg/day) did not show any effect on mortality. Of note, the use of hidroxycloroquine did not show significant improvement in OM. Other variables associated with higher mortality in this subset analysis of severely ill patients were; (iii) age > 70 years (OR 2.54, 95% CI 1.2–5.2, p = 0.011); (iv) uncontrolled hematological disease (OR 3.5, 95% CI 1.6–7.5, p = 0.001); (v) platelet count ≤ 20 × 10^9^/L (OR, 5.66, 95% CI 1.44–22, p = 0.013); (vi) platelet count 21–0  × 10^9^/L (OR 3.3, 95% CI 1.2–9.2, p = 0.021); (vii) history of arterial hypertension (OR, 2, 95% CI 1.3–3.2, p = 0.002); and (viii) CRP > 20 mg/dL (OR 2.7, 95% CI 1.1–6.5, p = 0.029).

## Discussion

We report herein a real-life experience with COVID-19 diagnosed in a large number of Hematology units in Spain over a two-month period during the first pandemic wave of the SARS-CoV-2 infection. Our results show an overall mortality rate at day 45 after diagnosis of 29% (27% COVID-19-related according to the treating physicians). Of note, a lower mortality was seen in recipients of a SCT (including allo-SCT recipients) as compared to non-SCT patients.

For its simplicity and the ability to classify cases into moderate or severe cases of pneumonia, we used the COVID-19 stages classification, as developed by Siddiqi et al. [15], and as expected found that stage IIB (pneumonia with acute respiratory failure) significantly correlated with higher mortality (shown in different study groups in Fig. 1a–d). This easy-to-implement classification could be of value for future observational studies and for the design of future randomized clinical trials. Additionally, we found that hypertension, lymphopenia and high CRP predicted for the development of stage IIB COVID-19. Although hypertension and lymphopenia were not found to independently increase mortality in our study, both variables have been shown to be strong predictors of mortality in the general population and we can thus postulate that with larger patient numbers they would also have had an impact on survival. On the other hand, higher mortality was linked with age > 70 years, uncontrolled hematological malignancy, baseline poor performance status, baseline severe neutropenia and a high CRP. With these RFs, we built a simple prognostic score based on the presence of 0–1, 2 or > 2 RFs, which clearly segregated the day 45 mortality into 3 groups. This segregation was obvious in the overall population [low risk (18% mortality), moderate risk (35% mortality) and high-risk (54% mortality); Fig. 2a], and also when tested separately in patients with pneumonia (Fig. 2b), in patients with uncontrolled or controlled underlying disease (Figs. 2c, d) and in SCT recipients as well as non-SCT patients (Fig. 2e, f). The lowest mortality was observed in patients with controlled hematological disease and without RFs (7%) in contrast to that observed in patients with uncontrolled hematological disease with > 2 RFs (80%). Of course, as with any risk score obtained form a single study, its validation and improvement in independent patient cohorts is required before one can suggest that it should be used to better predict the outcome of COVID-19 in patients with hematological malignancies.

Characteristics of COVID-19 symptoms in hematological patients share similarities with the general population [20], with fever, dry cough, fatigue and diarrhea being the most common initial signs/symptoms of infection. Systemic symptoms, however, were more common than URTD symptoms, as has also been reported in the general population. Compared to non-immunosuppressed patients [3], SARS-CoV-2 shedding in the upper airway seemed longer in our series (median of 24 days) with long-lasting shedding (> 21 days) in a significant proportion of studied cases (55%). The immunodeficiency driven by hematological malignancies and/or their treatment may explain a longer time for virus clearance. In the general population, low circulating B cell counts have been correlated with prolonged viral shedding [21]. In spite of the immunosuppressed status, the proportion of asymptomatic patients (8%) in our cohort was not irrelevant. Although our study was not designed to identify asymptomatic SARS-CoV-2 infections in our patients, it is obvious that these do exist, even if possibly at a lower rate than in the general population (reported to be approximately 30% of all infections) [22–26]. These observations support the recommendations for screening of SARS-CoV-2 infection in asymptomatic hematological patients before any scheduled treatments, including a planned SCT procedure, at least while the incidence in the community remains high.

In contrast with infections by other CARVs in hematological patients [27], the proportion of patients who develop LRTD when infected by SARS-CoV-2 appeared to be very high (79%), albeit this propensity for causing pneumonia has been well described in the general population [3]. In fact, the RFs we found for the development of severe COVID-19 pneumonia (stage IIB) were similar to those reported in the general population [3]. Hypertension [3, 4, 28, 29], lymphopenia [30–33] and high blood levels of markers of inflammation (including CRP) [34–36] are among these reported RFs. Due to the lack of data in up to half of our patients, we were unable to analyze the impact of other inflammatory/procoagulant biomarkers, such as ferritin, IL-6 and D-dimer on the risk of developing severe pneumonia and death, although they did show a clear trend in univariate analysis (as shown in Table 2).

The day 45 overall and COVID-19-related mortality in this series (29% and 27%, respectively) was comparable to other series of hematological patients [10–12], apparently higher than patients with solid tumors (13%) [37] and much higher than in the general population (2.3%) [38]. Somewhat surprising, however, was the lower mortality observed in SCT recipients, a patient group usually linked to the highest risk of death from opportunistic infections, including those caused by other CARV. However, patients who receive a SCT, especially an allo-SCT, are by definition younger and healthier than the overall onco-hematological patients. In fact, in our cohort we observed that most of conditions associated with higher overall mortality in multivariate analysis (i.e. older age, hypertension, uncontrolled hematological disease and high levels of CRP) were overrepresented in the non-SCT cohort, which may explain in part the lower mortality observed in SCT recipients. The fact that all patients with hematological malignancies were registered in the current study, without the strict inclusion criteria used in prospective treatment protocols and, especially, in clinical trials and for receiving a SCT, creates a much more real-life scenario. Indeed, clinicians are well accustomed to seeing elderly patients with many comorbidities who are far from being candidates to the standard-of-care for their AML, MDS, lymphoma, myeloma and other diseases, and patient-specific treatment decisions must commonly be made. In such real-life setting, COVID-19 mortality was mainly driven by advanced age, poor performance status, uncontrolled disease status (not being at least in a partial remission), presence of marrow or immune failure (severe neutropenia, and lymphopenia in the univariate analysis) and having a high baseline and/or COVID-19 induced systemic inflammatory state (identified in the multivariate analysis by high CRP, although high levels of IL-6 and ferritin showed a strong trend in univariate analysis). In contrast, the broad range of hematological malignancies included did not allow us to study the impact of this infection in different diagnoses.

We decided to evaluate the effect of several treatments on mortality only in patients who developed very severe COVID-19 and found that having received azithromycin (AZT) or low-dose corticosteroids were independently associated with lower mortality. Obviously, many clinicians have been using macrolides to treat COVID-19 off-label, without any robust evidence of safety or effectiveness. Our results suggest that the use of azithromycin at least did not have a deleterious impact on survival. In contrast, we found no impact of using hydroxychloroquine (HCQ). To what extend the lack of its benefit could be related to potential severe toxicities or insufficient antiviral activity remains to be determined [39]. Regarding the use of short courses of corticosteroids for COVID-19, prior reports have yielded conflicting results; both favorable [40–43] and detrimental effects [44, 45] have been found. A preliminary, unpublished analysis from a large, multicenter, randomized, open-label trial (RECOVERY study) showed that dexamethasone at 6 mg/day for 10 days reduced the mortality in patients with severe COVID-19 (defined as those who required supplemental oxygen) [46]. Our findings are in line with results from this trial, since 6 mg of dexamethasone is equivalent to 40 mgr of oral prednisone or 32 mgr of IV methylprednisolone, and the latter is within our definition of low-dose steroids (≤ 0.5 mg/kg/day methylprednisolone). However, our results should be interpreted with great caution and should not be used to support the use of either azithromycin or low-dose steroids in patients with hematological malignancies. Management of COVID-19 is a worldwide research priority, and the optimal treatment of our patients must be guided by the results of these international randomized clinical trials.

We acknowledge several limitations of this study such as its retrospective nature, the use of different PCR assays and the lack of complete inflammatory markers data in most cases. However, the large number of patients and the comparison between non-SCT and SCT recipients should be considered as its strengths, and a starting point for future larger and more disease-specific or treatment-specific observational studies. Additionally, our hematological COVID-19 database (see Fig. 1) mirrored national epidemiological data in SARS-CoV-2 during each week [47]. This fact suggests a low probability of bias in reporting hematological COVID-19 cases in our study.

## Conclusions

COVID-19 was severe in patients with hematological malignancies, and their survival was strongly correlated with the COVID-19 stage and patient and disease-related factors [older age, disease status, performance status, immune (neutropenia) status and systemic inflammation (high CRP)]. However, recipients of a SCT did not have a higher mortality.

## Data Availability

All data generated or analyzed during this study are included in this published article.
